# Supplementary material for: Proportion of stroke types in Madagascar: A tertiary-level hospital-based case series
Source: PLoS One. 2022 Oct 14;17(10):e0276199. doi: 10.1371/journal.pone.0276199 (PMC9565373; doi:10.1371/journal.pone.0276199)
Supplement: S1 File — (PDF) [file pone.0276199.s001.pdf]

Supporting Information

Proportion of stroke types in Madagascar: a tertiary-level hospital-based case series

Julia Riedmann[1], Andriamihaja Flavien Solonavalona[2], Adriamboahanginiaina Ravosoa Rakotozafy[2], Solofo Ralamboson[2], Matthias Endres[1,3,5,6,7,9], Bob Siegerink[3,4], Eberhard Siebert[8], Samuel Knauss ¶[1,9,10], Julius Valentin Emmrich ¶*[1,9,10]

[1]Department of Neurology, Charité - Universitätsmedizin Berlin, Germany

[2]Soavinandriana Military Hospital (CENHOSOA), Antananarivo, Madagascar

[3]Center for Stroke Research, Charité - Universitätsmedizin Berlin, Germany

[4]Department of Clinical Epidemiology, Leiden University Medical Center, Leiden University, Leiden, The Netherlands

[5]German Center for Neurodegenerative Diseases (DZNE), partner site Berlin, Germany

[6]German Centre for Cardiovascular Research (DZHK), partner site Berlin, Germany

[7]ExcellenceCluster NeuroCure, Berlin, Germany

[8]Institute of Neuroradiology, Charité - Universitätsmedizin Berlin, Germany

[9]Berlin Institute of Health, Berlin, Germany

[10]Heidelberg Institute of Global Health, Heidelberg University, Germany

*Corresponding author

E-mail: julius.emmrich@charite.de (JE)

¶ These authors contributed equally to this work.

S1 Table. Ultrasound imaging by stroke type.

	Total[a]	IS[a]
Total	223 (100)	128 (57.4)
Cardiac echo	54	50
no abnormalities	8 (14.8)	6 (12.0)
hypertensive cardiomyopathy	22 (40.7)	21 (42.0)
dilated cardiomyopathy	9 (16.7)	9 (18.0)
ischemic cardiomyopathy	9 (16.7)	9 (18.0)
mitral stenosis	5 (9.3)	5 (10.0)
other valvulopathy	2 (3.7)	2 (4.0)
pulmonary heart disease	3 (5.6)	3 (6.0)
intracavitary thrombus	2 (3.7)	2 (4.0)
aneurysm	3 (5.6)	3 (6.0)
arrythmia	2 (3.7)	2 (4.0)
other morphological pathologies	6 (11.1)	5 (10.0)
Carotid duplex sonography	22	22
no abnormalities	8 (36.4)	8 (36.4)
non-stenosing plaques	7 (31.8)	7 (31.8)
mildly-stenosing plaques	3 (13.6)	3 (13.6)
severe stenosis	1 (4.5)	1 (4.5)
unstable plaque[b]	1 (4.5)	1 (4.5)
intraluminal thrombus[c]	1 (4.5)	1 (4.5)
reduced carotid flow velocity[d]	1 (4.5)	1 (4.5)

IS = ischemic stroke; HS = hemorrhagic stroke

[a] number and % if not indicated otherwise

[b] unstable atherosclerotic lesion at risk of rupture described as “echolucent” or “predominantly echolucent” corresponding to types 1 and 2 in the Gray-Weale classification [1]

[c] intraluminal carotid artery thrombus at risk of embolization

[d] post-stenotic reduced blood flow velocity in carotid artery

References

1. Gray-Weale A.C., Graham J.C., Burnett J.R., Byrne K., Lusby R.J. Carotid artery atheroma: comparison of preoperative B-mode ultrasound appearance with carotid endarterectomy specimen pathology. J Cardiovasc Surg. 1988;29:676–681.
