# Supplementary material for: Proportion of stroke types in Madagascar: A tertiary-level hospital-based case series
Source: PLoS One. 2022 Oct 14;17(10):e0276199. doi: 10.1371/journal.pone.0276199 (PMC9565373; doi:10.1371/journal.pone.0276199)
Supplement: Attachment — Submitted filename: Response to Reviewers_rev2.pdf [file pone.0276199.s003.pdf]

Reviewer #1:

Please remove the terminologies: "unstable plaque" and "thrombus" from table S1. Actually, unstable plaque means plaque neovascularity or floating thrombus. I don't know if this is what the authors mean. I cannot understand what the authors mean by thrombus?

Response: We thank the reviewer for pointing this out and apologize for the imprecise terminology in the previous version. We acknowledge that by non-contrast enhanced duplex ultrasound the diagnostic accuracy to assess the stability of a plaque and the sensitivity to detect intraluminal thrombi is limited. We report the findings as recorded by the Malagasy physician performing the ultrasound examination. To better describe the findings, we added details of the classification used to assess stability and clarified the term “thrombus”.

The legend for “unstable plaque” now reads as:

“**unstable atherosclerotic lesion at risk of rupture described as “echolucent” or “predominantly echolucent” corresponding to types 1 and 2 in the Gray-Weale classification [1].”**

We omitted the term “thrombus” and specified to “intraluminal thrombus” which is also reflected in the legend now to read as:

“intraluminal carotid artery thrombus at risk of embolization”
